# Elucidating macrophage scavenger receptor 1’s mechanistic contribution as a shared molecular mediator in obesity and thyroid cancer pathogenesis via bioinformatics analysis

**DOI:** 10.3389/fgene.2024.1483991

**Published:** 2024-10-22

**Authors:** Fangjian Shang, Zhe Xu, Haobo Wang, Bin Xu, Ning Li, Jiakai Zhang, Xuan Li, Zhen Zhao, Xi Zhang, Bo Liu, Zengren Zhao

**Affiliations:** ^1^ Department of General Surgery, The First Hospital of Hebei Medical University, Shijiazhuang, Hebei, China; ^2^ Department of Urology, The First Hospital of Hebei Medical University, Shijiazhuang, Hebei, China; ^3^ Department of Radiology, The First Hospital of Hebei Medical University, Shijiazhuang, Hebei, China; ^4^ Department of Pharmacology, The Fourth Hospital of Hebei Medical University, Shijiazhuang, Hebei, China; ^5^ Department of General Surgery, The Fourth Hospital of Hebei Medical University, Shijiazhuang, Hebei, China

**Keywords:** obesity, thyroid carcinoma, macrophage scavenger receptor 1, bioinformatics analysis, machine learning

## Abstract

**Introduction:**

Obesity is a disease characterized by the excessive accumulation of fat. Concurrently, thyroid carcinoma (THCA) stands as the foremost endocrine malignancy. Despite the observed escalation in concurrent prevalence of both conditions, the underlying interconnections remain elusive. This indicates the need to identify potential biomarkers to predict the pathways through which obesity and THCA coexist.

**Methods:**

The study employed a variety of methods, including differential gene expression analysis, Weighted Gene Co-expression Network Analysis (WGCNA), and gene enrichment analysis. It was also supplemented with immunohistochemical data from the Human Protein Atlas (HPA), advanced machine learning techniques, and related experiments such as qPCR, to identify important pathways and key genes shared between obesity and THCA.

**Results:**

Through differential gene expression analysis, WGCNA, and machine learning methods, we identified three biomarkers (IL6R, GZMB, and MSR1) associated with obesity. After validation analysis using THCA-related datasets and biological experiments, we selected Macrophage Scavenger Receptor 1 (MSR1) as a key gene for THCA analysis. The final analysis revealed that MSR1 is closely related to the degree of immune cell infiltration in patients with obesity and THCA, suggesting that this gene may be a potential intervention target for both obesity and THCA.

**Discussion:**

Our research indicates that MSR1 may influence the occurrence and development of obesity and THCA by regulating the infiltration level of immune cells. This lays the foundation for future research on targeted therapies based on their shared mechanisms.

## 1 Introduction

Obesity is a significant public health issue characterized by abnormal or excessive accumulation of fat (Pamplona et al., 2022). This condition increases the risk of chronic diseases such as type 2 diabetes, cardiovascular diseases, and certain cancers (Biswas et al., 2017; Swinburn et al., 2011). Contemporary research has expanded from the traditional focus on obesity’s impact on metabolism and cardiovascular health to its complex interactions with various types of cancer, especially thyroid carcinoma (THCA) (Blériot et al., 2024; Schmid et al., 2015; Yuan et al., 2023). THCA is one of the most common endocrine malignancies, with its incidence continuously increasing worldwide (Lee et al., 2022; Jermain et al., 2022). Evidence suggests that obesity is a significant factor in the development and progression of THCA. Specifically, a meta-analysis of 21 studies involving 12,199 cases of THCA found that overweight individuals had a 25% increased risk of developing THCA compared to individuals of normal weight, while obese individuals had a 55% increased risk. This indicates a clear dose-response relationship between body weight and the risk of developing THCA, with the risk being substantially higher for those classified as obese (Schmid et al., 2015).

A growing body of research has initiated investigations into the mechanisms driving the obesity-THCA nexus. Studies have demonstrated an upregulation of Triiodothyronine (T3) expression in obese individuals, a pivotal hormone in the proliferation of thyroid cells. The heightened presence of T3 may be directly implicated in the oncogenesis of THCA among obese populations (Nannipieri et al., 2009; Hard, 1998). Additionally, a range of obesity-correlated biomarkers have been pinpointed as potential genetic markers for THCA’s early detection, prognosis assessment, or response to treatment. This includes the analysis of cytokines with pro-inflammatory and anti-inflammatory properties, such as TNF-α, IL-6, and IL-10. These cytokines are linked to obesity and are believed to influence THCA’s progression or therapeutic outcomes (Liu et al., 2012; Ozgen et al., 2009; Stassi et al., 2003; Iyengar et al., 2017). The MAPK signaling pathway is also posited as a critical conduit for thyroid carcinogenesis, where obesity-induced chronic inflammation may facilitate an excessive generation of reactive oxygen species (ROS), subsequently activating the MAPK pathway and leading to THCA’s advancement and invasiveness (Prete et al., 2020; Pérez-Torres et al., 2021; Nakamura et al., 2019). Nonetheless, the intricacies of the mechanisms fostering the obesity-THCA interrelation largely remain elusive (Ma et al., 2015).

In this study, we endeavor to uncover previously unidentified links between two diseases using bioinformatics and machine learning approaches. The advancements in bioinformatics have paved the way for the identification of potential biomarkers and their roles across various diseases (Akalin, 2006; Gu et al., 2019). Moreover, machine learning techniques are employed to delve into the pathological mechanisms and therapeutic targets at the genetic level across different diseases (Kumar et al., 2021). In our research, through an integrated analysis combining bioinformatics and machine learning, we identify shared key genes and molecular mechanisms between obesity and THCA, providing significant insights into the potential pathways through which obesity may facilitate the onset and invasion of THCA.

## 2 Methods and materials

### 2.1 Data collection and processings

Gene expression profiles were searched in the GEO database using “obesity” and “THCA” as keywords (Barrett et al., 2013). The inclusion criteria for the datasets were as follows: (1) gene expression analyses must include case and control groups. (2) Raw or processed data must be available for re-analysis. Three datasets were ultimately downloaded (GSE44000, GSE151839, GSE65144). GSE44000 (obesity: 7 cases; control: 7 cases) was conducted on the GPL6480 platform, while GSE151839 (obesity: 20 cases; control: 20 cases) and GSE65144 (THCA: 12 cases; control: 13 cases) were both conducted on the GPL570 platform. GSE44000 was used as the training set, and the external validation set was composed of GSE151839 and GSE65144 (Supplementary Table S1).

For gene expression analysis, the dataset’s series matrix files were log2 transformed, and then probes were mapped to their gene symbols using the annotation files of the respective platforms. This produced a gene matrix with gene column names and sample row names, which was used for subsequent analyses. For differential gene expression analysis, the “limma” package was used to perform linear modeling and empirical Bayes moderation of gene-wise variance.

### 2.2 Identification of differentially expressed genes

Differentially expressed genes (DEGs) between the obesity case group and the control group were obtained using the “limma” package in R software (version 4.2.2) (Leek et al., 2012). The differential expression was assessed using moderated t-tests, with cutoff criteria were set with an adjusted P-value of less than 0.05 and an absolute log fold change (|logFC|) greater than 1. Volcano plots were generated to highlight the differential expression of DEGs. Heatmaps were produced using the pheatmap package in R, based on the selected DEGs.

### 2.3 Weighted gene co-expression network analysis and selection of module genes

Using the “WGCNA” package, we conducted a Weighted Gene Co-expression Network Analysis (WGCNA) to identify key modules in obesity (GSE44000). WGCNA is a systems biology approach for constructing modules of co-expressed genes and exploring the correlation between genes and diseases (Alderden et al., 2018). Initially, the median absolute deviation (MAD) of each gene was determined, and the 50% of genes with the smallest MAD were removed. Secondly, samples with missing values and outliers were excluded using the Hclust function and the goodSamplesGenes function. Thirdly, adjacency was calculated using a “soft” thresholding power (β) derived from co-expression similarity, which was then transformed into a Topological Overlap Matrix (TOM) and its corresponding dissimilarity (1−TOM). Fourthly, a dendrogram of the TOM matrix was created using hierarchical clustering with average linkage, segregating similar gene expressions into different modules, with a minimum gene group size of (n = 100). Fifthly, the correlation between each module and the phenotype was assessed, where modules correlated with p< 0.05 were defined as key modules. Lastly, the network of characteristic genes was visualized. The statistical correlation between each module and the phenotype was evaluated using Pearson’s correlation coefficient.

### 2.4 Functional enrichment analysis

To identify shared biological processes and signaling pathways involved in the DEGs in obesity, we conducted Gene Ontology (GO) and Kyoto Encyclopedia of Genes and Genomes (KEGG) enrichment analysis using the “clusterProfiler” package in R. Additionally, we employed Gene Set Enrichment Analysis (GSEA) to uncover potential molecular mechanisms of key genes, utilizing the “clusterProfiler” package for analysis. Fisher’s exact test was used for GO and KEGG enrichment analyses, and enrichment analysis results with a statistical significance of P < 0.05 were considered significant in statistics, and were visualized using the Sangerbox platform (http://vip.sangerbox.com/).

### 2.5 Construction of PPI network and identification of hub genes

To observe the common functional features of DEGs, we constructed a protein-protein interaction (PPI) network using STRING (https://cn.string-db.org/), extracting PPI pairs with interaction scores greater than 0.15. Subsequently, the network was visualized using Cytoscape 3.9.1 (https://cytoscape.org). We utilized the CytoHubba plugin within Cytoscape to identify hub genes, employing eight ranking algorithms (Betweenness (BC), Eigenvector (EC), Closeness (CC), Degree (DC), Local Average Connectivity-based method (LAC), Network (NC), Subgraph (SC), Information (IC)). The ranks of the hub genes were determined by each algorithm, and a consensus ranking was obtained through intersection using Venn diagrams.

### 2.6 Machine learning

To further identify key genes for diagnosing obesity, two machine learning algorithms, Random Forest (RF) (Tai et al., 2019; Zhang et al., 2024; Ishwaran and Kogalur, 2010), and Support Vector Machine Recursive Feature Elimination (SVM-RFE) algorithm (Huang et al., 2018), were employed using the “randomForest” and “e1071″R packages (Austermann et al., 2014). RF can predict continuous variables and provide predictions with no apparent changes, offering the advantage of no variable condition restrictions. SVM-RFE, on the other hand, focuses on genes with high discriminatory power through fine selection. Feature importance in the Random Forest model was assessed through the Gini impurity index, whereas SVM-RFE used recursive elimination based on accuracy improvement. The intersection genes of RF and SVM-RFE are considered key genes for diagnosing obesity. To address potential overfitting, especially in the context of the small sample sizes used in this study, we will implement cross-validation techniques in future analyses. Additionally, we recognize the importance of utilizing more extensive performance metrics, such as precision, recall, and F1 score, to validate the robustness of our predictive models. This approach will enhance the reliability of our findings and provide a clearer understanding of the models’ performance. To construct a gene interaction network of the key genes and their neighboring genes, we utilized the GeneMania online database (http://www.genemania.org).

### 2.7 Screening and validation of key genes in THCA

To determine whether the key genes obtained from obesity are involved in THCA, we downloaded clinical data of THCA from The Cancer Genome Atlas database (https://tcga-data.nci.nih.gov/tcga/). This dataset comprised a total of 501 cases of THCA patients and 63 control patients. We used the t-test to compare gene expression levels between the case and control groups to assess for significant differences. Additionally, we evaluated the clinical diagnostic value of the key genes in THCA through Receiver Operating Characteristic (ROC) curve analysis. The statistical significance of the ROC curve was determined using the DeLong test. The area under the ROC curve (AUC) and its 95% confidence interval were calculated to quantify the diagnostic value of key genes for the disease, with an AUC >0.5 considered ideal diagnostic value (Zhou et al., 2021). Furthermore, we selected immunohistochemical images of key genes in THCA and normal tissues from the Human Protein Atlas (HPA) database (https://www.Proteinatlas.org/). These images were used to detect differential expression of key genes at the protein level.

### 2.8 Evaluation and correlation analysis of infiltrating immune cells

We utilized the “ggstatsplot” and “ggplot2″ packages to analyze the Spearman correlation between key genes and immune infiltrating cells in obesity, presenting the results graphically. Subsequently, we employed TIMER, EPIC, IPS, MCP-counter, xCELL, CIBERSORT, and QUANTISEQ algorithms to explore the relationship between Macrophage Scavenger Receptor 1 (MSR1) expression and immune infiltration in THCA. Spearman’s rank correlation coefficient was used to determine the relationship between MSR1 expression and immune cell infiltration. A statistical significance in immune cell infiltration was considered when P < 0.05 (Le et al., 2021).

### 2.9 Real-time quantitative RT-PCR

This study involved a total of 50 adult inpatients diagnosed with THCA. Tissue samples were collected from the cancerous regions of these patients and designated as the THCA group, while adjacent non-cancerous tissue samples were collected and designated as the Control group. The research protocol strictly adhered to the principles outlined in the Declaration of Helsinki, ensuring ethical handling of human tissues. Furthermore, the study was approved by the Clinical Research Ethics Committee of the First Hospital of Hebei Medical University. Prior to their participation, each patient provided informed consent by signing the necessary forms.

To ensure that the sample sizes used in the qPCR validation were sufficient to detect meaningful differences, we conducted a power analysis. This analysis was performed to determine the minimum number of samples required to achieve adequate statistical power (typically set at 0.8). The results of the power analysis indicated that our sample size of 50 patients was sufficient to detect significant differences in gene expression between the THCA and Control groups.

To extract total RNA from human liver tissue, the RNA isolation kit (RNAiso, Takara, San Jose, CA, United States) was utilized. In this experiment, the isolated RNA was dissolved in 20 mL of DEPC-treated water. Subsequently, reverse transcription was performed using the reverse transcription reagent kit (mL RT reagent kit with gDNA Eraser, Takara) and a thermal cycler (ἧ, Eppendorf, Hamburg, Germany). The resulting cDNA was used for qPCR detection, and amplification curves were generated using SYBR PremiexExTaqII from Takara. Statistical comparison of gene expression between THCA and Control groups was performed using a paired t-test, with P < 0.05 considered statistically significant. This experimental validation ensured the accuracy and reliability of the identified gene expression levels, reinforcing the robustness of our results. The primers used for quantitative PCR are as follows:

GAPDH forward - AAT​GGA​CAA​CTG​GTC​GTG​GAC;

GAPDH reverse - CCC​TCC​AGG​GGA​TCT​GTT​TG;

MSR1 forward - GTT​AGG​GGT​TTG​GAC​TGC;

MSR1 reverse - GAT​GTG​GCC​ACC​AAA​TAC.

### 2.10 Statistical analysis

All statistical analyses were conducted using the R statistical software. To assess whether the data complied with a normal distribution, the Shapiro-Wilk test was employed. Differential analyses of gene expression, immune cell infiltration, and other parameters were performed using appropriate statistical tests based on the normality assessment (such as t-tests for comparing two groups, analysis of variance (ANOVA) for comparing multiple groups, and chi-square tests for categorical variables). The significance threshold was set at a P-value less than 0.05. Correlation coefficients were computed to determine the relationship between biomarkers and clinical features, ensuring the integrity and validity of our results.

## 3 Results

### 3.1 Identification of DEGs in obesity

**FIGURE 1:**
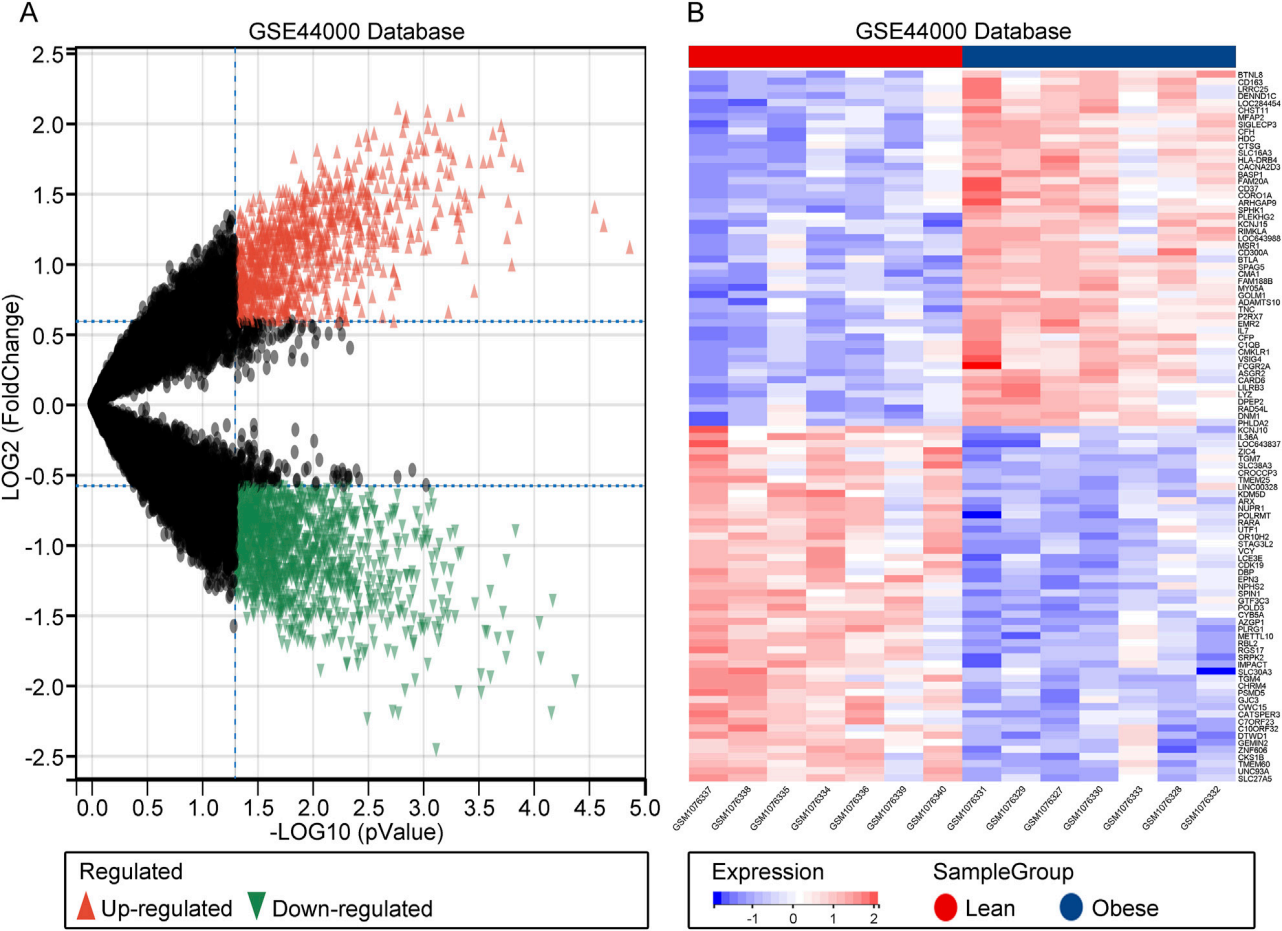
Identification of DEGs. **(A)** Heatmap of DEGs in GSE44000. **(B)** Volcano plot of DEGs in GSE44000.

The flow chart of this study is shown in Supplementary Figure S1. In GSE44000, a total of 2,131 DEGs were identified, including 894 upregulated genes and 1,237 downregulated genes. These DEGs were visualized in both heatmaps and volcano plots (Figures 1A, B). In summary, a comprehensive identification of DEGs in obesity has been achieved, providing a foundation for further analyses.

### 3.2 Identifying key modules in obesity

**FIGURE 2:**
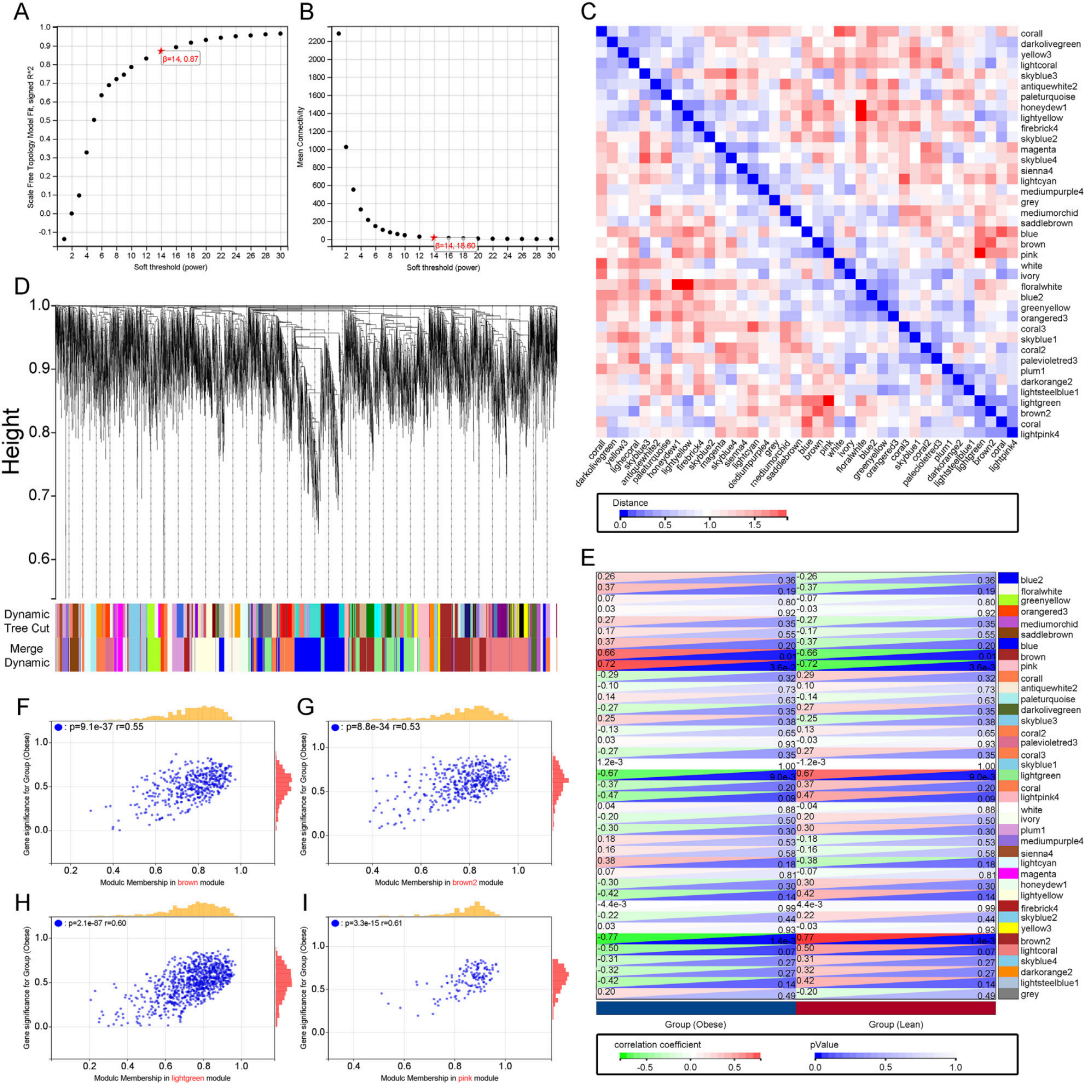
Construction of WGCNA Co-expression Network. **(A)**, **(B)** Soft thresholding power (β = 7) and scale-free topology fit index (R2). **(C)** Co-linearity heatmap of module eigengenes. Red indicates high correlation, blue indicates the opposite. **(D)** Display of original and merged modules under the clustering tree. **(E)** Heatmap of module-trait correlations. Red represents positive correlation, blue represents negative correlation. **(F–I)** MM vs. GS scatter plot of the brown module, pink module, light green module and brown2 module.

Using WGCNA analysis, a soft threshold of 14 was set in GSE44000 when R^2 = 0.87, ensuring biologically meaningful scale-free networks (Figures 2A, B). Subsequently, 39 modules were detected by merging strongly correlated modules with a clustering height cut-off of 0.25. The modified and merged modules were ultimately displayed under the clustering tree (Figure 2D). The correlation between modules was examined, revealing no significant associations between them (Figure 2C). Investigating the relationship between modules and clinical symptoms, the brown module showed a positive correlation with obesity (r = 0.66, p = 0.01) and a negative correlation with normal status (r = −0.66, p = 0.01). Similarly, the pink module exhibited a positive correlation with obesity (r = 0.72, p = 0.0036) and a negative correlation with normal status (r = −0.72, p = 0.0036), while the light green module showed a negative correlation with obesity (r = −0.67, p = 0.009) and a positive correlation with normal status (r = 0.67, p = 0.009). Additionally, the brown2 module displayed a negative correlation with obesity (r = −0.77, p = 0.0014) and a positive correlation with normal status (r = 0.77, p = 0.0014) (Figure 2E). Finally, clinically significant modules were identified, indicating a strong correlation with obesity in the MM *versus* GS scatter plots (Figures 2F–I). Consequently, all genes within these four modules were further examined. In conclusion, key modules related to obesity have been identified, highlighting potential biomarkers for further investigation.

### 3.3 Enrichment analysis of immune-related overlapping genes in obesity

**FIGURE 3:**
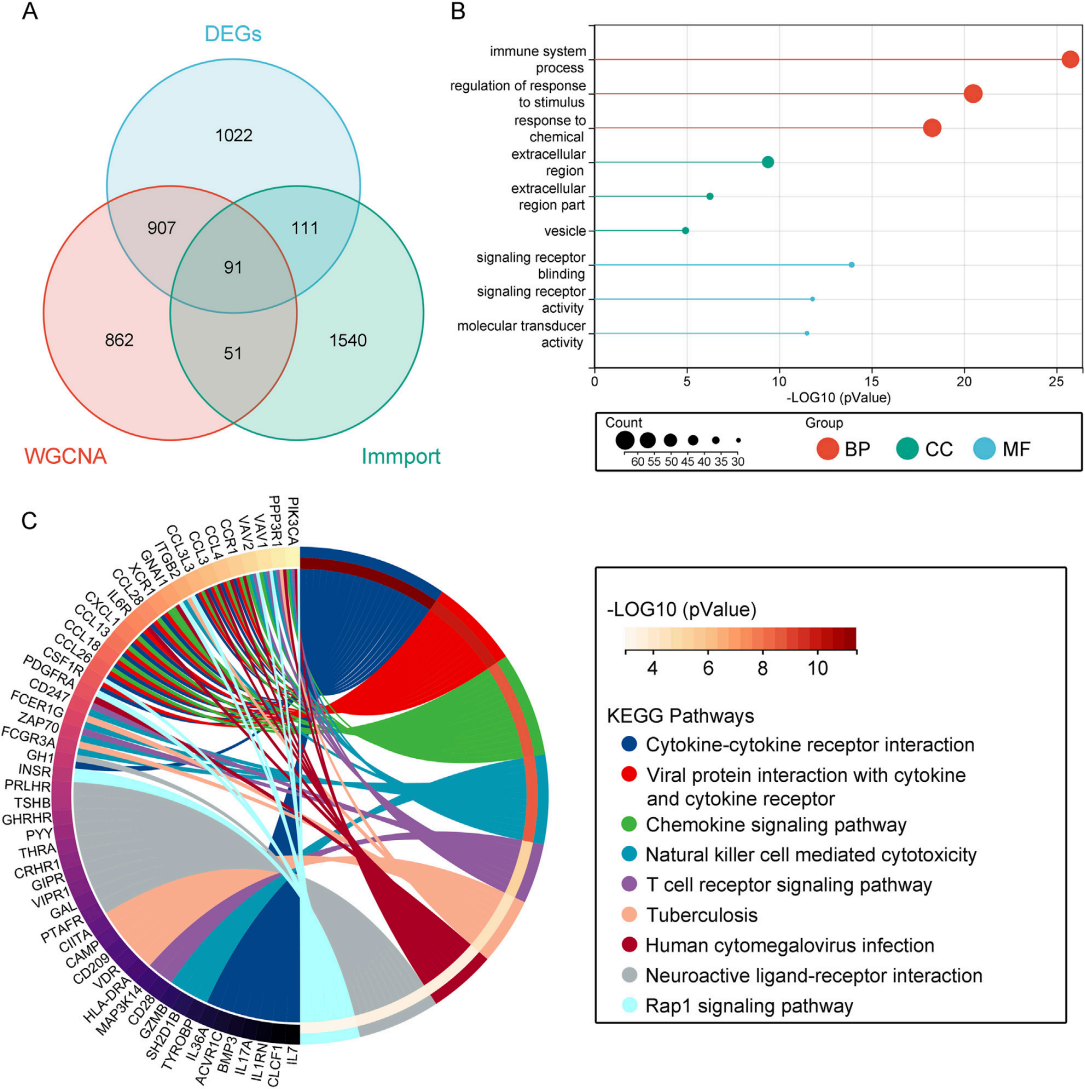
Enrichment Analysis of Immune-Related Overlapping Genes. **(A)** Venn diagram showing immune genes from the ImmPort database overlapped with key module genes and DEGs. **(B)** GO analysis. **(C)** KEGG analysis.

To acquire immune-related genes associated with obesity, we extracted 1793 immune-related genes from the ImmPort database. Utilizing a Venn diagram, we overlapped these genes with the DEGs obtained from the GEO database and the key module genes identified from WGCNA, revealing a total of 91 overlapping genes related to immunity (Figure 3A). Subsequently, functional analysis was conducted to understand the biological functions of these overlapping genes. GO enrichment analysis indicated that biological processes (BP) were primarily enriched in the regulation of stimulus response, response to chemicals, and immune system processes. Molecular functions (MF) were associated with signal receptor binding, signal receptor activity, and molecular transducer activity. Cellular components (CC) enrichment was related to extracellular region, extracellular region part, and vesicle (Figure 3B; Supplementary Table S2). In the KEGG analysis, pathways such as cytokine-cytokine receptor interaction, chemokine signaling pathway, interaction of viral proteins with cytokines and cytokine receptors, natural killer cell mediated cytotoxicity, neuroactive ligand-receptor interaction, tuberculosis, human cytomegalovirus infection, T cell receptor signaling pathway, and Rap1 signaling pathway were found to be relevant (Figure 3C; Supplementary Table S3). Thus, this analysis elucidates the significant immune-related pathways and processes involved in obesity.

### 3.4 Identifying key genes in obesity through PPI network and machine learning

**FIGURE 4:**
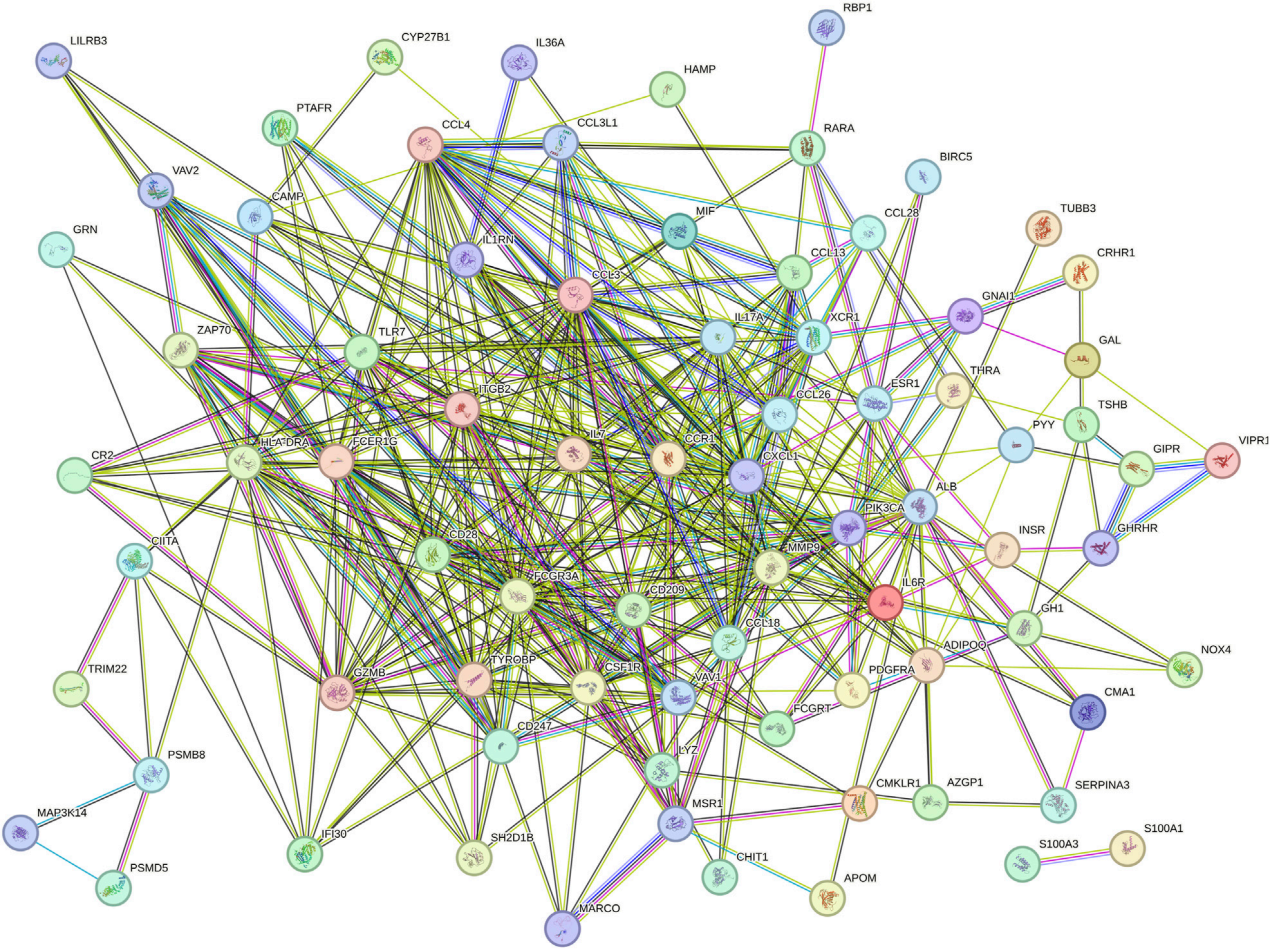
PPI network of overlapping DEGs.

**FIGURE 5:**
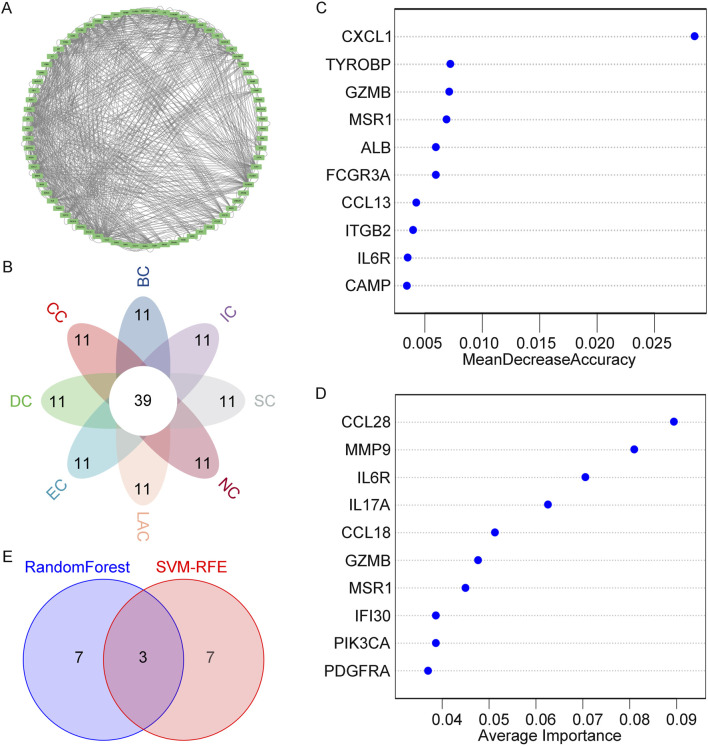
Identification of Hub Genes and Key Genes. **(A)** Detailed view of network nodes in Cytoscape. **(B)** Hub genes identified by eight centrality algorithms in Cytoscape. **(C)** Biomarker selection based on the RF algorithm. **(D)** Biomarker selection based on SVM-RFE. **(E)** Venn diagram showing the intersection of key genes obtained through both algorithms.

To analyze the PPI network of overlapping genes, we utilized STRING, as shown in Figure 4. The PPI network comprised 91 nodes and 423 edges. Subsequently, we further refined the understanding of the network using Cytoscape, highlighting central genes as key nodes (Figure 5A). The cytoHubba plugin in Cytoscape was employed to identify hub genes, utilizing eight ranking algorithms (BC, EC, CC, DC, LAC, NC, SC, IC), selecting the top 50 genes from each method, and determining 39 common hub genes via a petal plot (Figure 5B; Supplementary Table S4). Following this, to further identify key genes from the hub genes, we employed two machine learning algorithms: RF and SVM-RFE. RF can predict continuous variables and provide predictions with no apparent variance, offering the advantage of no variable condition limitations (Figure 5C). SVM-RFE, on the other hand, focuses on fine selection, targeting genes with high discriminative power (Figure 5D). Through a Venn diagram, we found three key genes from the intersection of these two methods: GZMB, MSR1, and IL6R (Figure 5E). These findings highlight the identification of crucial genes involved in the pathophysiology of obesity.

### 3.5 Expression and Validation of Key Genes in obesity

**FIGURE 6:**
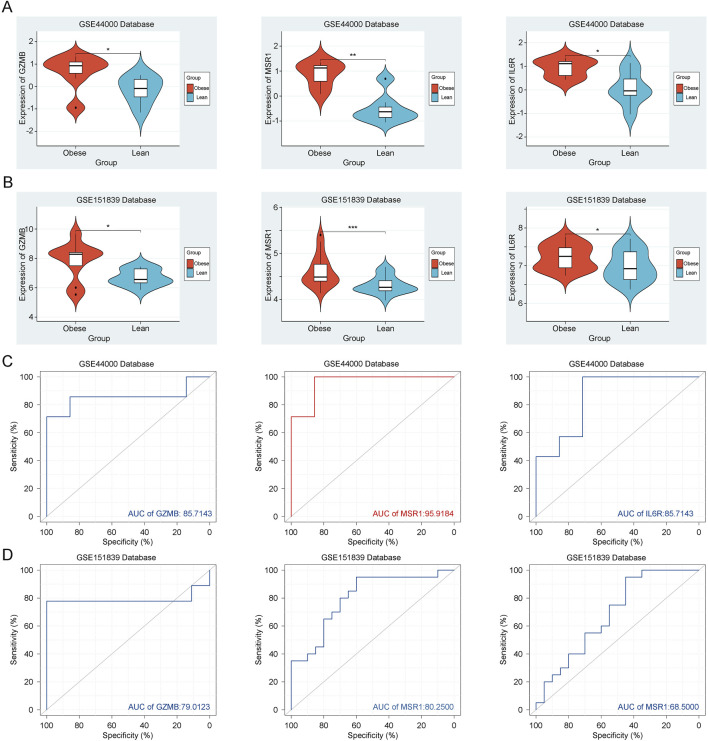
Expression and Validation of Key Genes. **(A)** Expression of GZMB, MSR1 and IL6R in the training group GSE44000. **(B)** Expression of GZMB, MSR1 and IL6R in the validation group GSE151839. **(C)** ROC curve of GZMB, MSR1 and IL6R in the training group GSE44000. **(D)** ROC curve of GZMB, MSR1 and IL6R in the validation group GSE151839.

Initially, in the training dataset GSE44000, we observed significant upregulation of GZMB, MSR1, and IL6R in adipose tissues of the obesity group (Figure 6A). The established ROC curve results also indicate high diagnostic value of GZMB (AUC: 0.857), MSR1 (AUC: 0.959), and IL6R (AUC: 0.857) for obesity (Figure 6C). Subsequently, we selected an additional dataset, GSE151839, associated with obesity as the validation group. In the validation group, GZMB, MSR1, and IL6R were similarly significantly upregulated in adipose tissues of the obesity group (Figure 6B). The ROC curve results also demonstrate high diagnostic value of GZMB (AUC: 0.790), MSR1 (AUC: 0.959), and IL6R (AUC: 0.857) for obesity (Figure 6D). This confirms that these three key genes are associated with obesity and should be further examined in subsequent analyses. Overall, these key genes are validated as reliable biomarkers for obesity diagnosis and merit further exploration.

### 3.6 Analysis of immune infiltration and GSEA analysis of key genes in obesity

Due to the recognized role of immune-inflammatory responses as the foremost regulatory factors in obesity, the CIBERSORT method was employed to elucidate the immune modulation in obesity. The heatmap reveals that GZMB upregulates the expression levels of monocytes, dendritic cells, and eosinophils in obesity, MSR1 upregulates the expression levels of macrophages and natural killer cells in obesity, and IL6R upregulates the expression levels of dendritic cells in obesity (Supplementary Figure S2A). Through GSEA analysis, we found that in obesity, GZMB is primarily involved in antigen processing and presentation, natural killer cell-mediated cytotoxicity, cell adhesion molecules (CAMs), leukocyte transendothelial migration, cytokine-cytokine receptor interaction, chemokine signaling pathway, JAK-STAT signaling pathway, and FCεRI signaling pathway (Supplementary Figure S2B). MSR1 is primarily involved in natural killer cell-mediated cytotoxicity, focal adhesion, and FCεRI signaling pathway (Supplementary Figure S2C). IL6R is primarily involved in lysosome, leukocyte transendothelial migration, B cell receptor signaling pathway, and FCγR-mediated phagocytosis (Supplementary Figure S2D). In summary, the analysis underscores the immune-related pathways and gene functions that may contribute to obesity pathogenesis.

### 3.7 Analysis of immune infiltration and GSEA analysis of key genes in obesity

**FIGURE 7:**
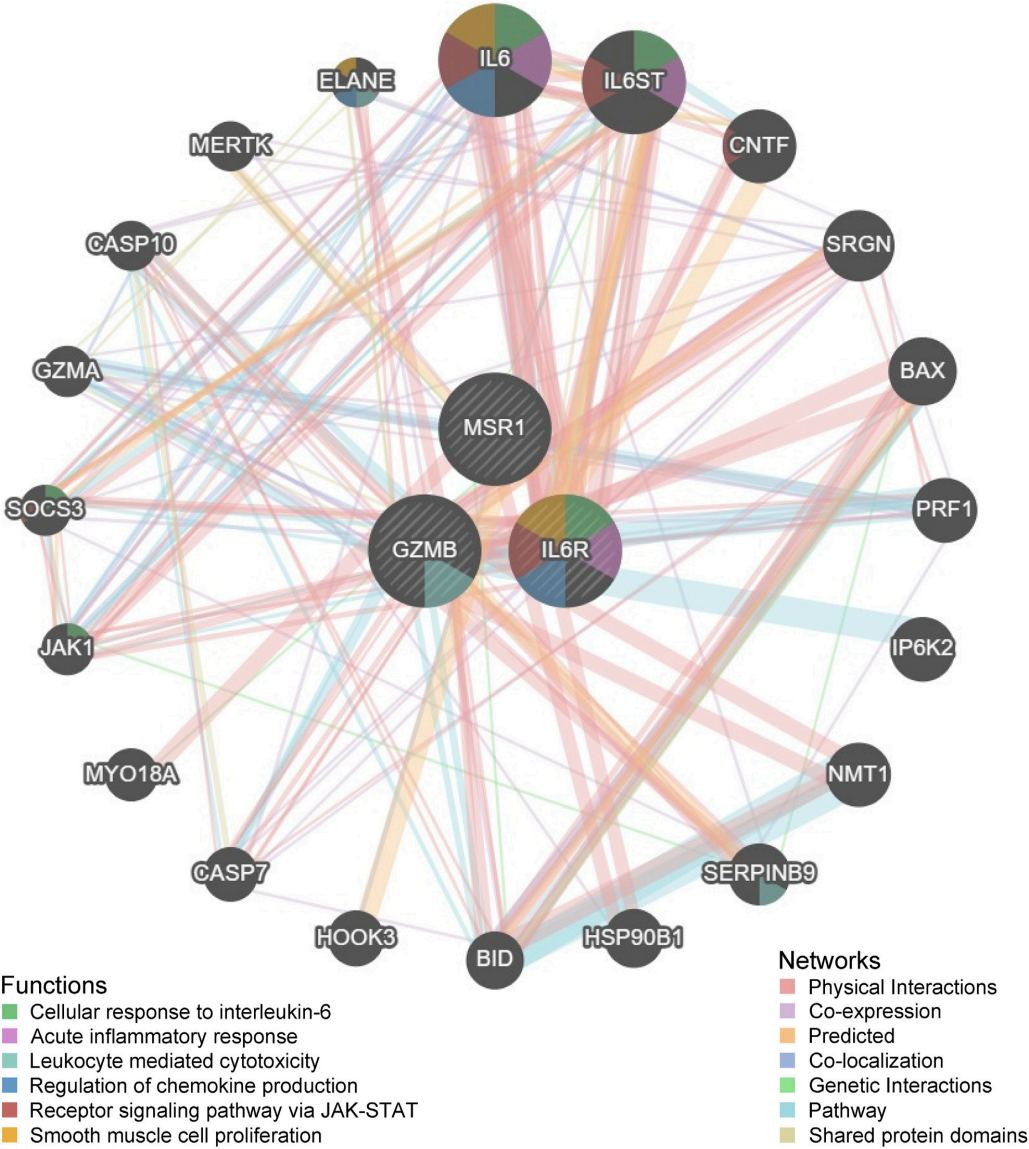
Functional analysis of three key genes and twenty interacting genes conducted through the GeneMania database.

To further investigate the functions of key genes, three key genes and twenty interacting genes underwent functional analysis, and a co-expression network was constructed using the GeneMania database. The three key genes are represented in the inner circle, while the outer circle represents genes connected to the key genes. Figure 7 indicates that these genes are mainly enriched in cellular responses to interleukin-6, acute inflammatory responses, leukocyte-mediated cytotoxicity, regulation of chemokine production, receptor signaling through the JAK-STAT pathway, and smooth muscle cell proliferation. Overall, these findings suggest that MSR1 may serve as a significant biomarker linking obesity and thyroid cancer.

### 3.8 Key gene screening and validation in THCA

As immune response is crucial not only in obesity but also in THCA, we examined whether there is any connection between these two diseases using the key genes. In the TCGA dataset, GZMB did not show significant differential expression between normal and thyroid tumor tissues. In contrast, MSR1 was significantly upregulated in THCA. IL6R exhibited marked downregulation in tumor tissues (Supplementary Figure S3A). Which was confirmed by paired analysis (Supplementary Figure S3B). To validate the reliability of the key genes, we selected another GEO dataset, GSE65144, related to THCA as the validation group. In the validation dataset, we observed significant upregulation of MSR1 in THCA tissues, while GZMB and IL6R did not show significant differential expression (Supplementary Figure S3C). ROC curve results also demonstrated that MSR1 (AUC = 0.923) is a perfect diagnostic indicator with extremely high diagnostic accuracy for THCA, while GZMB (AUC = 0.679) and IL6R (AUC = 0.551) were particularly suboptimal, with diagnostic capabilities close to random levels for THCA (Supplementary Figure S3D). This confirms MSR1 as an important biomarker regulating both obesity and THCA. These findings reinforce the role of MSR1 in tumor progression and underscore its potential as a biomarker for thyroid cancer.

### 3.9 MSR1 Immunohistochemical Staining and Enrichment Analysis in THCA

**FIGURE 8:**
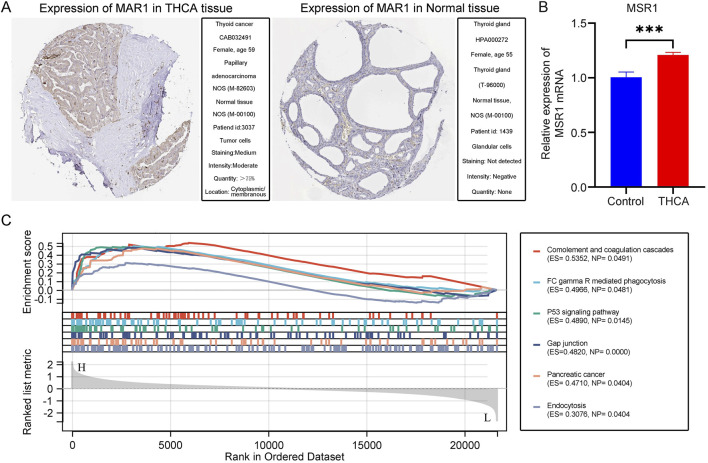
Immunohistochemical Staining and Enrichment Analysis of MSR1. **(A)** Immunohistochemical staining of MSR1 in normal and THCA tissues according to the HPA database. **(B)** A quantitative analysis of MSR1 mRNA transcription levels was conducted in THCA tissue samples (n = 50) and adjacent non-cancerous tissues (n = 50). **(C)** Major enrichment pathways of MSR1 in THCA.

To validate the selected key gene, we conducted immunohistochemical analysis of MSR1 in THCA tissues using the HPA database. The results revealed a significant increase in MSR1 expression in THCA tissues compared to the control group (Figure 8A). Subsequently, we employed qPCR to further quantitatively analyze the mRNA levels of MSR1 in human THCA tissues. Consistent with our previous findings, MSR1 expression was significantly upregulated in the THCA group compared to the adjacent non-cancerous control group (Figure 8B). Finally, through GSEA, we found that MSR1 was primarily enriched in complement and coagulation cascades, FCγR-mediated phagocytosis, the p53 signaling pathway, gap junctions, and pancreatic cancer in THCA (Figure 8C). Overall, these findings indicate that MSR1 is significantly upregulated in THCA tissues and is involved in critical pathways related to tumor progression and immune response.

### 3.10 MSR1 Immunoinfiltration Analysis in THCA

**FIGURE 9:**
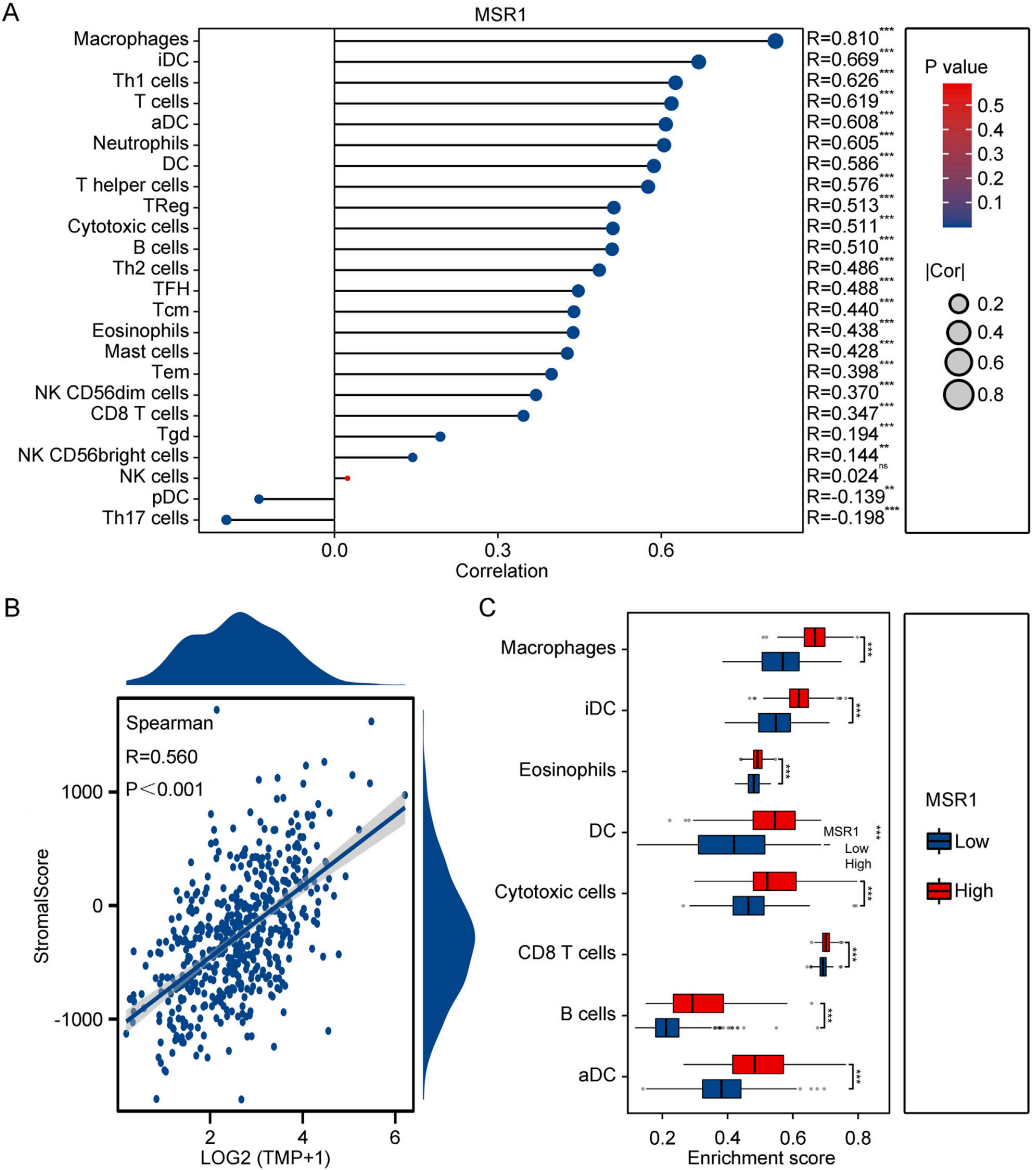
Immunoinfiltration Analysis of MSR1 in THCA. **(A)** Correlation of MSR1 with immune cells in THCA and normal samples. **(B)** Spearman correlation between MSR1 expression and StromalScore. **(C)** Enrichment scores of immune cells and pathways between high and low MSR1 expression in THCA.

To gain further insights into the role of MSR1 in tumor immune responses, we employed the CIBERSORT method to elucidate the immune modulation of MSR1 in THCA. The results demonstrated that MSR1 was significantly upregulated in THCA, particularly in macrophages, dendritic cells, and T cell subtypes (Figure 9A). Moreover, the positive correlation between MSR1 levels and StromalScore further supported the potential role of MSR1 in the formation and maintenance of the tumor microenvironment, particularly with components associated with the tumor stroma (Figure 9B). Furthermore, the enrichment scores of immune cell infiltration indicated that in the high MSR1 expression group compared to the low MSR1 expression group, most immune cell types showed higher enrichment, particularly in aDCs, CD8 T cells, cytotoxic cells, and eosinophils, suggesting a correlation between high MSR1 expression and the abundance of these immune cells (Figure 9C). Lastly, to further understand the role of MSR1 in tumor immune responses, we utilized seven algorithms including TIMER, EPIC, IPS, MCP-counter, xCELL, CIBERSORT, and QUANTISEQ to assess the relationship between MSR1 expression and the infiltration of immune cells at different levels. The TIMER algorithm revealed significant upregulation of B cells, CD4^+^ T cells, neutrophils, and myeloid dendritic cells by MSR1 (Supplementary Figure S4A). The EPIC algorithm showed significant upregulation of neutrophils and macrophages by MSR1 (Supplementary Figure S4B). The IPS algorithm demonstrated significant upregulation of endothelial cells and stem cells by MSR1 (Supplementary Figure S4C). The MCP-counter algorithm indicated significant upregulation of T cells, B cells, and NK cells by MSR1 (Supplementary Figure S4D). The xCELL algorithm indicated significant upregulation of CD8^+^ T cells and cytotoxic T cells by MSR1, among others (Supplementary Figure S4E). The CIBERSORT algorithm demonstrated upregulation of various immune cells by MSR1 (Supplementary Figure S4F). Lastly, the QUANTISEQ algorithm showed significant upregulation of B cells, M1, and M2 macrophages by MSR1 (Supplementary Figure S4G). These analyses highlight the critical role of MSR1 in modulating immune cell infiltration in THCA, emphasizing its potential as a therapeutic target for enhancing anti-tumor immunity.

## 4 Discussion

Obesity, as a major global public health concern, is increasingly being linked to the pathogenesis of various cancers, including THCA (Huang et al., 2024; Yu et al., 2024). The simultaneous rise in the incidence rates of obesity and THCA suggests a potential connection between the two (Masone et al., 2021). Emerging evidence indicates that obesity not only predisposes individuals to THCA but also influences its progression, thereby highlighting the need for a deeper investigation into this relationship. In this study, we employed bioinformatics approaches to explore the molecular associations between obesity and THCA. Through in-depth analysis of GSE44000, we identified 2,131 DEGs. WGCNA analysis revealed key module genes closely associated with obesity. Following identification of overlapping genes between DEGs, module genes, and those in the Immport database using Venn diagrams, we further identified hub genes in obesity through PPI network analysis, and precisely identified GZMB, MSR1, and IL6R as biomarkers in obesity using machine learning methods. Validation of these three key genes in the THCA-related dataset revealed that MSR1 is the only gene exhibiting significantly different expression in THCA tissues. Furthermore, our qPCR results also showed that the transcription level of MSR1 in THCA tumor tissues was significantly increased compared to adjacent normal tissues. These findings are consistent with our previous results, indicating that MSR1 may play an important role in the development and progression of THCA. Subsequent immune infiltration analysis revealed that MSR1 can modulate specific types of immune cells to influence the immune microenvironment in obesity and THCA, thereby impacting the development of both diseases. Thus, we hypothesize that MSR1 is a crucial factor underlying the link between obesity and THCA, providing novel targets for future therapeutic strategies for these two diseases. In addition, the other significant genes identified in our analysis, particularly IL6R and GZMB, also warrant further exploration. Their roles in the obesity-THCA axis could provide additional insights into the molecular mechanisms at play and enhance the overall understanding of disease pathology. Future studies should aim to investigate the functional roles of IL6R and GZMB, as this could reveal their contributions to obesity and THCA and potentially identify new therapeutic targets.

The MSR1 protein, encoded by the MSR1 gene, is a transmembrane protein primarily expressed by macrophages. Initially discovered on chromosome 19, it was later found to be present on all chromosomes except the mitochondrial genome (Wei et al., 2024). Recent studies have shown significant inhibition of ovarian and pancreatic cancer development in mice lacking MSR1 (Neyen et al., 2013). This suggests that MSR1 may play a role in macrophage-induced tumor activation and act as a molecular switch regulating gene expression (Ji et al., 2022). In this study, we found that MSR1 is upregulated in obesity, associated with inflammation-related immune cells such as macrophages and natural killer cells, while in THCA, it enhances the activity of macrophages, dendritic cells, and T cell subsets. This finding highlights the critical role of MSR1 in regulating disease-specific immune cell infiltration. Furthermore, GSEA analysis revealed that MSR1 affects key immune regulatory pathways in obesity and THCA, such as natural killer cell-mediated cytotoxicity, cytokine-cytokine receptor interaction, and the JAK-STAT signaling pathway, further confirming the multifunctionality of MSR1 in disease progression and its importance as a potential therapeutic target. Given these insights, the role of MSR1 in modulating the immune response presents a significant opportunity for developing targeted therapies. For instance, pharmacological agents that enhance or inhibit MSR1 activity could be explored as potential treatments for obesity-related THCA. Additionally, understanding the mechanisms by which MSR1 influences immune cell behavior could inform the design of immunotherapies that improve clinical outcomes for patients with these conditions. Therefore, MSR1’s role as a therapeutic target not only has implications for understanding disease mechanisms but also provides a pathway for clinical applications aimed at obesity and THCA treatment.

In this study, we integrated various advanced techniques, including WGCNA, PPI network analysis, and machine learning algorithms, to identify the key gene MSR1 and assess its diagnostic value for obesity and THCA patients, ensuring the depth and breadth of the research findings. Additionally, we validated our findings through multiple independent datasets, enhancing the credibility and applicability of the discoveries. However, a major limitation of this study is the lack of laboratory validation, particularly regarding direct evidence of the specific biological functions and mechanisms of action of MSR1 in disease progression. Therefore, to enhance the translational potential of this research, it will be essential to strengthen our understanding of the functional role of MSR1 through experimental validation and to explore its potential applications in the treatment of obesity and THCA.

## 5 Conclusions

This study, through the integration of bioinformatics analysis and machine learning, has discovered that MSR1 can influence the occurrence and development of obesity and THCA by modulating the infiltration of immune cells. This provides new diagnostic and therapeutic strategies for obesity-related THCA. However, it is important to note that this study has certain limitations and shortcomings, primarily stemming from the lack of a more profound investigation into the mechanisms underlying MSR1’s role in the interplay between obesity and THCA. Despite these limitations, our study establishes a robust theoretical framework that paves the way for future research to further elucidate the relationship between obesity and THCA.

## Data Availability

The original contributions presented in the study are included in the article/Supplementary Material, further inquiries can be directed to the corresponding authors.
