# Supplementary material for: Elucidating macrophage scavenger receptor 1’s mechanistic contribution as a shared molecular mediator in obesity and thyroid cancer pathogenesis via bioinformatics analysis
Source: Front Genet. 2024 Oct 22;15:1483991. doi: 10.3389/fgene.2024.1483991 (PMC11534819; doi:10.3389/fgene.2024.1483991)
Supplement: Supplementary file 1 [file DataSheet1.PDF]

*Supplementary Material*

| Group | GEO Data set | Platform | Obesity/THCA | Normal |
|---|---|---|---|---|
| Analysis set | GSE44000 | GPL6480 | 7 | 7 |
| Validation set | GSE151839 | GPL570 | 20 | 20 |
| Validation set | GSE65144 | GPL570 | 12 | 13 |

**Supplementary Table 1.** Information on microarray datasets obtained from Gene Expression Omnibus.

| Ontology | Term | Description |
|---|---|---|
| BP | GO:0048583 | Regulation of response to stimulus |
| BP | GO:0042221 | Response to chemical |
| BP | GO:0002376 | Immune system process |
| CC | GO:0005576 | Extracellular region |
| CC | GO:0044421 | Extracellular region part |
| CC | GO:0031982 | Vesicle |
| MF | GO:0005102 | Signaling receptor binding |
| MF | GO:0038023 | Signaling receptor activity |
| MF | GO:0060089 | Molecular transducer activity |

**Supplementary Table 2:** GO enrichment significance of overlapping genes.

| Ontology | ID | Description |
|---|---|---|
| KEGG | hsa04060 | Cytokine-cytokine receptor interaction |
| KEGG | hsa04062 | Chemokine signaling pathway |
| KEGG | hsa04061 | Viral protein interaction with cytokine and cytokine receptor |
| KEGG | hsa04650 | Natural killer cell mediated cytotoxicity |
| KEGG | hsa04080 | Neuroactive ligand-receptor interaction |
| KEGG | hsa05152 | Tuberculosis |
| KEGG | hsa05163 | Human cytomegalovirus infection |
| KEGG | hsa04660 | T cell receptor signaling pathway |
| KEGG | hsa04015 | Rap1 signaling pathway |

**Supplementary Table 3:** KEGG pathways of overlapping genes.

| Rank | Ranking methods in the CytoHubba plugin | | | | | | | |
|---|---|---|---|---|---|---|---|---|
| | Betweenness(BC) | Eigenvector(EC) | Closeness(CC) | Degree(DC) | Local Average Connectivity-based method(LAC) | Network(NC) | Subgraph(SC) | Information(IC) |
| 1 | ALB | FCGR3A | FCGR3A | FCGR3A | CCL3 | FCGR3A | FCGR3A | FCGR3A |
| 2 | HLA-DRA | ALB | ALB | CCL3 | FCGR3A | CCL3 | CCL3 | ALB |
| 3 | FCGR3A | CCL3 | CCL3 | CCL4 | CCL4 | ITGB2 | CCL4 | CCL3 |
| 4 | MMP9 | CXCL1 | ITGB2 | ITGB2 | CSF1R | CCL4 | ITGB2 | ITGB2 |
| 5 | PSMB8 | ITGB2 | MMP9 | IL17A | ITGB2 | ALB | IL17A | MMP9 |
| 6 | CCR1 | CCL4 | CCR1 | CSF1R | GZMB | IL17A | CSF1R | CCR1 |
| 7 | ESR1 | MMP9 | CCL4 | CXCL1 | CXCL1 | CXCL1 | CXCL1 | CCL4 |
| 8 | CXCL1 | CSF1R | IL17A | MMP9 | IL17A | FCER1G | MMP9 | IL17A |
| 9 | PYY | IL17A | CXCL1 | CCR1 | TLR7 | MMP9 | CCR1 | CXCL1 |
| 10 | GNAI1 | GZMB | FCER1G | GZMB | CD28 | CCR1 | GZMB | FCER1G |
| 11 | CCL3 | CCR1 | TYROBP | ALB | FCER1G | TYROBP | ALB | TYROBP |
| 12 | ITGB2 | TLR7 | CSF1R | FCER1G | IL7 | CSF1R | FCER1G | CSF1R |
| 13 | IL17A | FCER1G | GZMB | TLR7 | CCR1 | GZMB | TLR7 | GZMB |
| 14 | GZMB | TYROBP | IL7 | IL7 | MMP9 | IL7 | IL7 | IL7 |
| 15 | CCL4 | CD28 | TLR7 | TYROBP | TYROBP | TLR7 | TYROBP | TLR7 |
| 16 | GH1 | IL7 | CD28 | CD28 | CD247 | CD28 | CD28 | CD28 |
| 17 | TYROBP | CD209 | HLA-DRA | CD209 | CD209 | IL1RN | CD209 | HLA-DRA |

| | | | | | | | | |
|---|---|---|---|---|---|---|---|---|
| 18 | PIK3CA | ESR1 | IL1RN | HLA-DRA | IL6R | ZAP70 | HLA-DRA | IL1RN |
| 19 | RARA | IL1RN | CCL18 | IL1RN | ZAP70 | CCL18 | IL1RN | CCL18 |
| 20 | CIITA | IL6R | CD209 | IL6R | IL1RN | CD247 | IL6R | CD209 |
| 21 | ADIPOQ | HLA-DRA | ZAP70 | CCL18 | XCR1 | HLA-DRA | CCL18 | ZAP70 |
| 22 | FCER1G | ADIPOQ | PIK3CA | XCR1 | CCL18 | XCR1 | XCR1 | PIK3CA |
| 23 | TLR7 | PDGFRA | XCR1 | ZAP70 | ALB | CD209 | ZAP70 | XCR1 |
| 24 | CSF1R | LYZ | ESR1 | CD247 | HLA-DRA | IL6R | CD247 | ESR1 |
| 25 | IL1RN | ZAP70 | IL6R | CCL26 | VAV1 | VAV1 | CCL26 | IL6R |
| 26 | INSR | PIK3CA | VAV1 | CCL13 | CCL13 | CCL13 | CCL13 | VAV1 |
| 27 | IL7 | XCR1 | CD247 | CCL3L1 | CCL26 | CCL26 | CCL3L1 | CD247 |
| 28 | LYZ | CCL18 | ADIPOQ | PIK3CA | VAV2 | PIK3CA | PIK3CA | ADIPOQ |
| 29 | GHRHR | VAV1 | CCL26 | VAV1 | CCL3L1 | VAV2 | VAV1 | CCL26 |
| 30 | GAL | FCGRT | CCL13 | MSR1 | MIF | CCL3L1 | MSR1 | CCL13 |
| 31 | XCR1 | CAMP | MSR1 | LYZ | PIK3CA | MIF | LYZ | MSR1 |
| 32 | IL6R | MIF | CCL3L1 | ESR1 | SH2D1B | SH2D1B | ESR1 | CCL3L1 |
| 33 | VAV1 | CD247 | LYZ | MIF | CR2 | ADIPOQ | MIF | LYZ |
| 34 | CAMP | MSR1 | CAMP | ADIPOQ | MSR1 | MSR1 | ADIPOQ | CAMP |
| 35 | CCL18 | CCL3L1 | VAV2 | CR2 | LYZ | CR2 | CR2 | VAV2 |
| 36 | THRA | CCL26 | IFI30 | CAMP | ADIPOQ | ESR1 | CAMP | IFI30 |
| 37 | CD28 | CCL13 | FCGRT | FCGRT | CCL28 | CAMP | FCGRT | FCGRT |

| | | | | | | | | |
|---|---|---|---|---|---|---|---|---|
| 38 | MSR1 | IFI30 | PDGFRA | VAV2 | CAMP | LYZ | VAV2 | PDGFRA |
| 39 | TSHB | PYY | MIF | IFI30 | IFI30 | CCL28 | IFI30 | MIF |
| 40 | GIPR | VAV2 | CR2 | PDGFRA | ESR1 | IFI30 | PDGFRA | CR2 |
| 41 | CD209 | GNAI1 | SH2D1B | SH2D1B | FCGRT | FCGRT | SH2D1B | SH2D1B |
| 42 | PDGFRA | CCL28 | CCL28 | CCL28 | PTAFR | PTAFR | CCL28 | CCL28 |
| 43 | FCGRT | RARA | RARA | PTAFR | MARCO | MARCO | PTAFR | RARA |
| 44 | ZAP70 | CR2 | CIITA | MARCO | LILRB3 | LILRB3 | MARCO | CIITA |
| 45 | IFI30 | PTAFR | GNAI1 | LILRB3 | PDGFRA | PDGFRA | LILRB3 | GNAI1 |
| 46 | CCL26 | GH1 | GH1 | RARA | RARA | PSMB8 | RARA | GH1 |
| 47 | CCL13 | SH2D1B | PYY | CIITA | NOX4 | NOX4 | CIITA | PYY |
| 48 | CCL28 | LILRB3 | GHRHR | PYY | CIITA | CIITA | PYY | GHRHR |
| 49 | CCL3L1 | CMA1 | TSHB | CMA1 | GH1 | RARA | CMA1 | TSHB |
| 50 | CD247 | CMKLR1 | PSMB8 | GNAI1 | INSR | GH1 | GNAI1 | PSMB8 |

**Supplementary Table 4:** Hub genes identified using the Cytohubba plugin of Cytoscape.

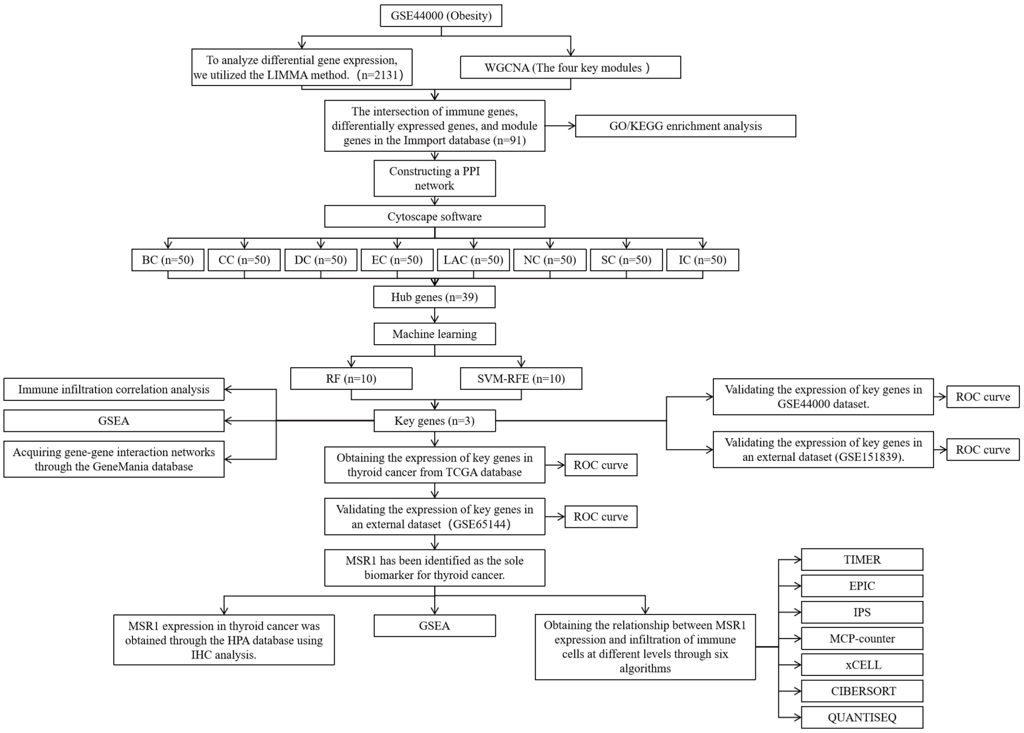


**Supplementary Figure 1:** Flow chart of the study.

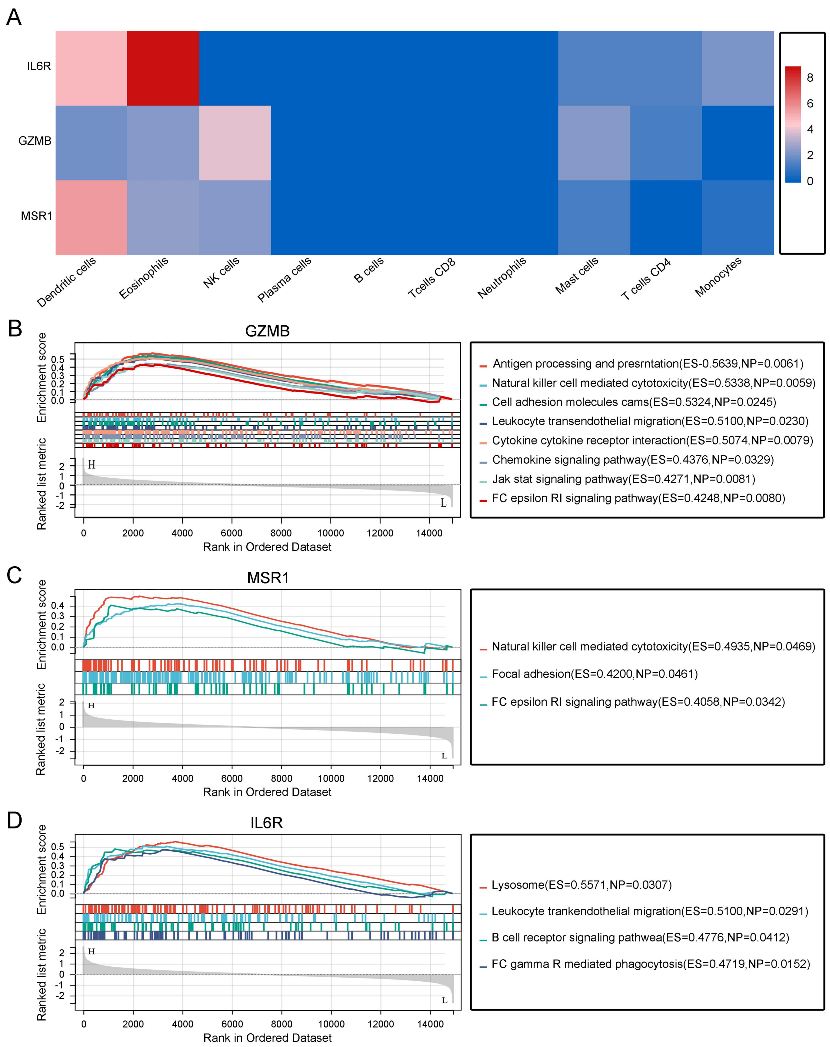


**Supplementary Figure 2:** Immune Infiltration Analysis and GSEA Analysis of Key Genes. (A) Heatmap illustrating the involvement of key genes in immune cell regulation. (B) Major enrichment pathways of GZMB. (C) Major enrichment pathways of MSR1. (D) Major enrichment pathways of IL6R.

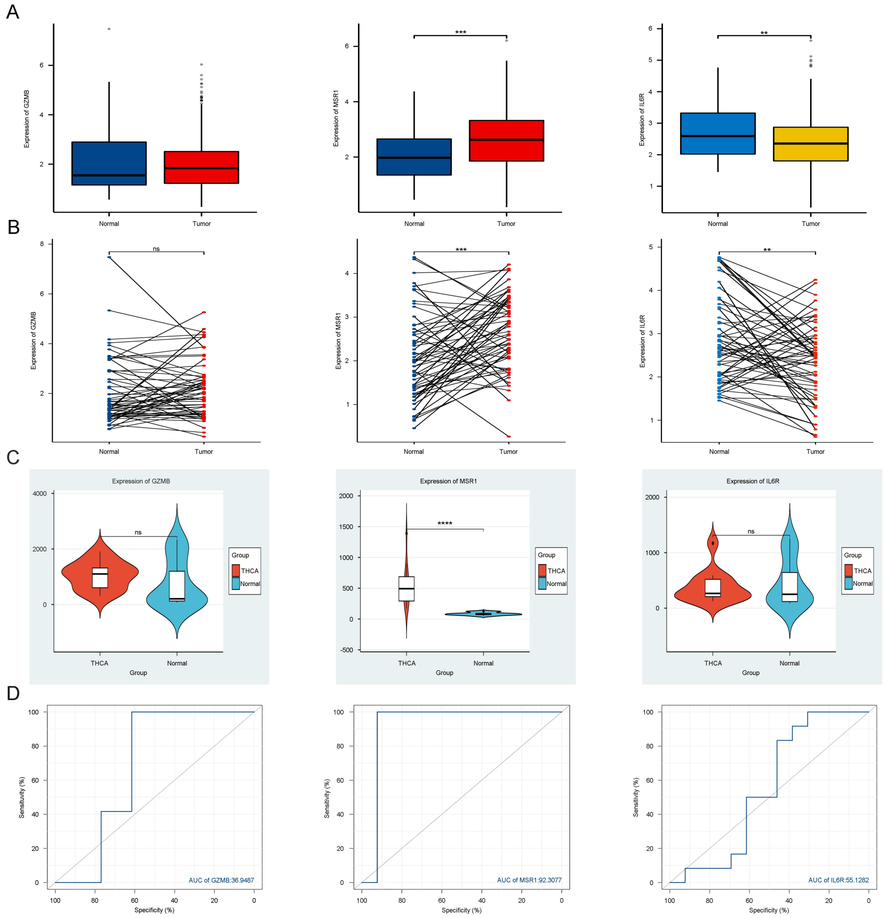


**Supplementary Figure 3:** Screening of Key Genes in Thyroid Cancer. (A) Expression of GZMB, MSR1 and IL6R in thyroid cancer from the TCGA database. (B) Differential expression analysis of GZMB, MSR1 and IL6R between normal and thyroid cancer tissues. (C) Expression of GZMB, MSR1 and IL6R in the validation group GSE65144. (D) ROC curve of GZMB, MSR1 and IL6R in the validation group GSE65144.

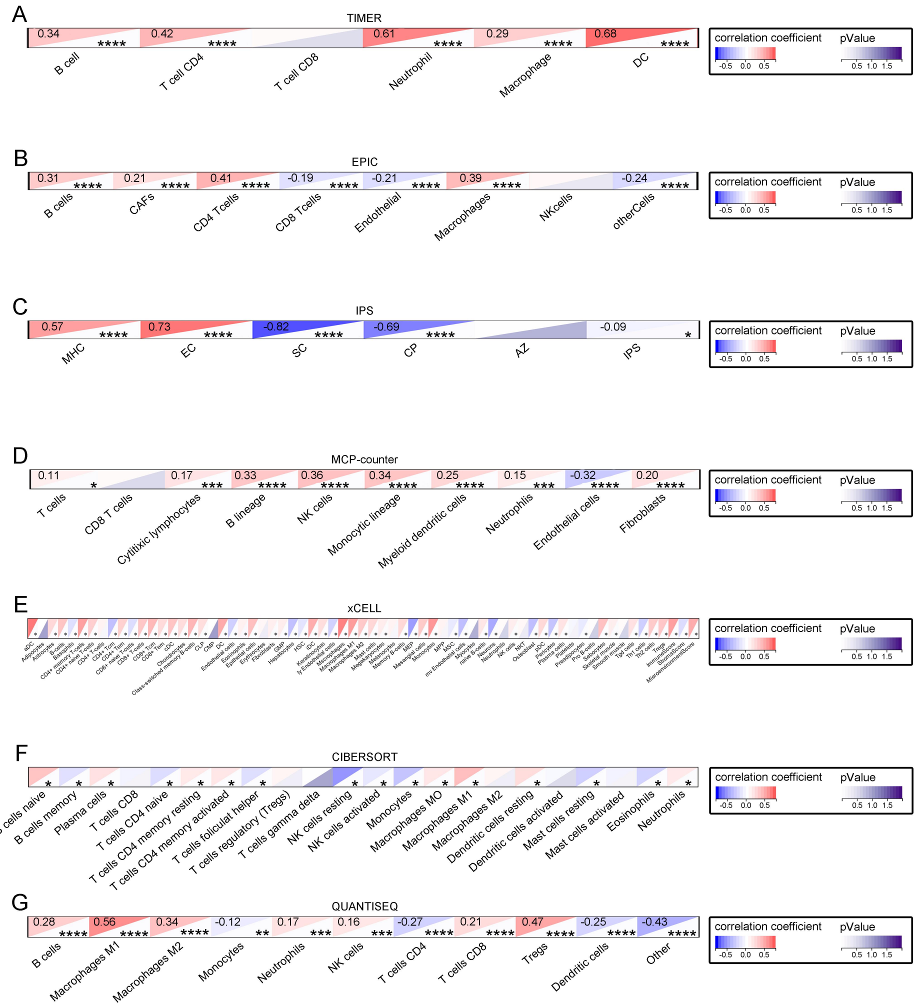


**Supplementary Figure 4:** Role of MSR1 in Tumor Immune Response. (A) TIMER immune score. (B) EPIC immune score. (C) IPS immune score. (D) MCP-counter immune score. (E) xCELL immune score. (F) CIBERSORT immune score. (G) QUANTISEQ immune score.
